# Gender-Specific Association Between Sleep Duration and Body Mass Index in Rural China

**DOI:** 10.3389/fendo.2022.877100

**Published:** 2022-05-31

**Authors:** Lin Ren, Li Chang, Yijun Kang, Yaling Zhao, Fangyao Chen, Leilei Pei

**Affiliations:** ^1^ Department of Epidemiology and Health Statistics, School of Public Health, Xi’an Jiaotong University Health Science Center, Xi’an, China; ^2^ Department of Quality Control, Xi’an Fourth Hospital, Xi’an, China; ^3^ The First Department of General Surgery, Shaanxi Province Tumor Hospital, Xi’an Jiaotong University Health Science Center, Xi’an, China

**Keywords:** sleep duration, BMI, obesity, gender-specific, rural China

## Abstract

**Background:**

This study aimed at investigating the association of sleep duration with body mass index (BMI) by gender among adult residents in rural Hanzhong of Shaanxi province, Northwest China.

**Methods:**

A two-level stratified random cluster sampling method was used to select adult residents between the ages of 18 and 80 years. All information including sociodemographic characteristics and lifestyles was collected by face-to-face interview with a structured questionnaire. According to standard methods, trained staff were responsible for anthropometric measurements using calibrated instruments in an empty room. By gender, both ordinary least square regression (OLS) and quantile regression (QR) were used to analyze the relationship between sleep time and BMI controlling for other confounders. The restricted cubic splines with five knots were further used to express the potentially non-linear association between sleep time and BMI.

**Results:**

A total of 3,017 eligible participants were included in the study. After controlling for confounding factors including sociodemographic characteristics and lifestyles, OLS regression did not indicate any significant association of sleep duration with BMI among men and women. Among men, it was clear that there is an inverse U-shaped relationship between sleep time and BMI beyond the 66.0th percentile (BMI ≥24). Among women, quantile regression presented a significant U-shaped relationship between BMI and sleep duration. According to the restricted cubic splines, the women who sleep for approximately 9 h had the lowest BMI, and when sleep duration approached approximately 7 h among men, their BMI would be the highest.

**Conclusions:**

The U-shaped and inverse U-shaped relationships between sleep duration and BMI were clearly observed for women and men, respectively, in our study. The identification of potentially relevant modifiable risk factors may provide better preventive approaches to obesity.

## Introduction

The rapidly increasing prevalence of obesity has recently become a major public health problem worldwide (1, 2). Studies have indicated that overweight and obesity are strongly associated with the increased risk for cardiovascular disease, hypertension, type 2 diabetes, and certain cancers (3). Due to the economic transition and introduction of the western lifestyle, the prevalence of obesity in the developing countries is rising even faster than the historical experience of the developed regions (4–6). It is reported that the yearly growth rates of overweight and obesity in Asia, North Africa, and Latin America are two to five times greater than those in the USA (4). The China Nutrition and Health Survey shows that the prevalence of overweight and obesity increased by 38.6% and 80.6%, respectively, in 1992–2002, with a higher growth rate in rural areas than in urban areas (7). In Northwest China with a lower economic level compared to other regions of China, the prevalence of overweight and obesity has reached 27.8% and 5.7%, respectively, in our previous studies (8).

This obesity epidemic coincided with the change of sleep duration in modern society. With economic development, interestingly, today we have more leisure activities and modern lifestyles, and restricted or excessive sleep duration has become prevalent (9). For example, a recent survey conducted in the USA showed that the prevalence of sleepers reporting less than 6 h of sleep was 29.2%, and approximately 8% of adult respondents reported sleeping 9 or longer hours (10, 11). A similar change has also occurred in China in the past decades, and approximately a third of the adults sleep less than 7 h or more than 9 h per night (12). Mounting evidence from laboratory studies with animals and humans suggests a mechanistic link between restricted or excessive sleep duration and increasing body weight (13–15). Since sleep is associated with neuroendocrine function and alteration of appetite-regulating hormone, as well as specific behavior, such as low physical activity and low consumption of fruit and vegetables, it is not a surprise that the changes in sleep duration increase the risk of obesity (16, 17).

Considering that men and women have different lifestyles, hormonal influences, and social concepts, the sleep duration–obesity relationship in adults is possibly less consistent by gender. A meta-analysis by Itani et al. revealed that short sleep was associated with a significant increase in both sexes in obesity, but long sleep duration was associated with a significant increase in incident obesity only among women (13, 14). Results from the Korea National Health and Nutrition Examination Survey suggested that there was an increased risk for obesity for short and long sleep duration compared with optimal sleep duration in women but not in men (18). These clues indicate the possibility that sleep duration may influence the development of obesity by gender.

From a public health perspective, the identification of the association between sleep duration and obesity may bring about the development of better preventive approaches to obesity. However, little is known about gender disparities on sleep duration and the effect on the association with obesity. Therefore, we explore whether the relationship between sleep duration and obesity is gender-specific. In 2010, we conducted an epidemiological survey of obesity in rural residents of ages 18 to 80 years in Hanzhong, Shaanxi province, in Northwest China. On the basis of the survey data, for now, we aim to investigate the sleep duration and its association with body mass index (BMI) by gender among adult residents in the surveyed areas. To the best of our knowledge, this is the first study to examine the influence of sleep duration on BMI among adults in rural Northwest China.

## Materials and Methods

### Study Design and Participants

A population-based epidemiological survey of obesity was conducted from October to November in 2010 in rural areas of Hanzhong, Shaanxi province, in Northwest China. According to the cross-sectional design of the study, a two-level stratified random cluster sampling method was used to select adult residents between the age of 18 to 80 years. There are nine townships in the study area and about 17 (15 to 36) villages in each township. Sampling procedures are presented in detail as follows. Firstly, nine strata were determined according to the township. Then one village was randomly chosen from each township. Using residential registration data, all the available and eligible adults (approximately 400 participants) in the chosen villages were informed and invited to participate in the survey. Finally, we expected that a total of approximately 3,600 participants could be recruited for the survey.

### Data Collection

A face-to-face interview was conducted with a family questionnaire to collect the participants’ sociodemographic characteristics and lifestyles. In our study, the family questionnaire used for data collection was grouped into two parts as follows: (1) characteristics questionnaire, including age, gender, education years, marital status, the resources of household income, housing condition, type of vehicle, communication tools, and the availability of clean water, and (2) individual lifestyles questionnaire, including sleep duration every day (h/night), farming frequency (times/week), hours of TV viewing, smoking status, drinking alcohol frequency (times/week), intake frequencies of sweets, fatty meat, and fried foods (times/week). To assess sleep duration (h/night), the participants were asked ‘‘How many hours do you on average sleep during the night?’’ Physical exercise was also self-reported in terms of the frequency of vigorous physical activities (times/week), including running, aerobics, heavy yard work, or anything else that causes large increases in breathing or heart rate.

According to standard methods, trained staff were responsible for anthropometric measurements using calibrated instruments in an empty room in every village. When standing height was measured using a non-stretchable tape (214 Road Rod™, USA), participants were required to take off their shoes, put the feet together, and place the arms on their sides. Moreover, we also made sure their heels, buttocks, and upper back were also in contact with the wall when the measurement was made. Ultimately, the maximum distance from the floor to the highest point on the head facing directly ahead was obtained as standard standing height (accurate to 1 mm). After participants removed all heavy clothes and shoes, weight was measured using a calibrated electronic scale (Tanita HD-305, Japan) with an accuracy of 0.1 kg.

### Quality Control

In the survey, all questionnaires were designed by the Xi’an Jiaotong University Health Science Center. A pilot study was carried out to pretest the questionnaire and procedures, and then the detailed interviewer guides were developed. The investigation team was established for questionnaire surveys and anthropometric measures, consisting of 10 to 12 members and one supervisor from the Xi’an Jiaotong University Health Science Center. As soon as each questionnaire was completed, the supervisors were under obligation to detect errors and/or missing values in the questionnaire. Informed consent was obtained from each participant at the start of the survey. This study was conducted in accordance with the Declaration of Helsinki and was reviewed and approved by the Human Research Ethics Committee of the Xi’an Jiaotong University Health Science Center (Number: 2002001).

### Study Variables

In the current study, the daily sleep duration of the participants was obtained using a quantitative measure. Because of its ease of implementation, body mass index (BMI; kg/m^2^) was used as an index of overweight and obesity in the study. Studies had shown that at the same level of BMI, Chinese people had a higher percentage of body fat and a higher prevalence of many obesity-related diseases, compared to European/North American populations. The Working Group on Obesity in China (WGOC) had suggested that the BMI-based definition of overweight or obesity for the Chinese population should be lower than that for the European or North American population (19, 20). In this study, weight status categories were defined as follows: <18.5 kg/m^2^ (underweight), 18.5 kg/m^2^ ≤ BMI ≤ 23.9 kg/m^2^ (normal weight), 24.0 kg/m^2^ ≤ BMI ≤ 27.9 kg/m^2^ (overweight), and BMI ≥ 28.0 kg/m^2^ (obesity).

To assess the economic status of respondents, a wealth index was developed by means of the principal component analysis. The index combined information on an inventory of household assets or facilities, including the resources of household income, housing condition, type of vehicle, communication tools, and the availability of clean water. In this process, only the first of the components produced with maximum variance was extracted to represent the household wealth (21). This index was categorized into tertiles indicating the poorest, medium, and wealthiest households. Farming and physical exercise frequencies were divided into three categories: none (<1 time/week), sometimes (1–4 times/week), and often (≥5 times/week). Smoking in the study was also classified into three groups, including current smoker (participants who were still smoking at the visit), never smoker (participants who at the survey stated that they had never smoked), and ex-smoker (it had been more than 6 months since participants stopped smoking). The intake frequencies of sweets, fatty meat, and fried foods were also divided into three subgroups: none (<1 time/week), sometimes (1–7 times/week), and often (≥7 times/week). Drinking alcohol frequency (no and ≥1 time/week) was also included in the study.

### Statistical Analysis

A database was established using EpiData Version 3.0 (Odense, Denmark), and data were double-entered to reduce data entry errors. Data were expressed as numbers or percentages and presented as mean with standard deviation of the mean. Firstly, we explored the distribution of sociodemographic characteristics and lifestyles across different cutoffs of BMI by gender. Based on previous studies (22, 23), moreover, we also divided sleep duration into three subgroups, including <7 h (short), 7–9 h (normal), and >9 h (long). Then, we also assessed the differences in the sociodemographic characteristics and lifestyles across three sleep duration subgroups. In the study, all univariate comparisons across subgroups were evaluated using ANOVA, χ^2^, and Kruskal–Wallis as appropriate.

Since too high or too low of BMI will lead to completely different health outcomes, the effect of sleep duration on BMI may be heterogeneous in the populations of different BMI categories. Due to the ordinary least square (OLS) model reporting only the central tendency of the marginal effects of covariates on the dependent variable, it may provide limited information on the relationship between sleep time and BMI. By comparison, quantile regression (QR) employed in this study enables the investigation of covariates on BMI changes across the entire distribution of BMI by relaxing the constant marginal effects of the explanatory variables under the OLS specification.

In the study, the relationship between sleep time and BMI was investigated using the regression specification of the different conditional quantiles of BMI (quantile = 0.02, 0.05, 0.25, 0.50, 0.68, 0.71, 0.80, 0.85, 0.95, 0.97, 0.99) after adjustment for sociodemographic characteristics and lifestyle among the participants. For comparison, we also ran the ordinary least squared (OLS) model to estimate the mean regression to compare risk factors of the mean BMI. All regression analyses were conducted separately for men and women. The interaction effects of sleep duration and gender on BMI were tested using quantile regression in advance, whereas the statistically significant conclusion could not be obtained. Considering the non-linear relationship between sleep duration and BMI, squared sleep duration (SleepSQ) was entered into the study.

To further validate the findings, we used restricted cubic splines with five knots to express the potentially non-linear association between sleep time and BMI. We did a corresponding sensitivity analysis to exclude underweight participants to reduce the influence of underweight on the results. All statistical analyses were performed using R 4.1.2, and significance was achieved from statistical tests when P < 0.05.

## Results

### Characteristics of Participants

In the survey, we enrolled 3,030 participants between the ages of 18 and 80 years with a response rate of 84.17%. Because of 13 participants with missing BMI, a total of 3,017 eligible participants were included in the final study. In the analysis process, we further excluded the missing values of covariates (4 for age, 27 for education years, 31 for sleep time, 117 for TV viewing time, 17 for marriage, 22 for physical activity, 46 for farming, 17 for frequency of sweets intake, 18 for frequency of fat intake, 19 for frequency of fried foods intake, 10 for smoking, 13 for alcohol consumption). Because of the different sample sizes between men and women (1,048 vs. 1,969) in the study, the cutoff of obesity in men was the 94.1th quantile, and the value in women was the 94.4th quantile. A significant gender difference in sociodemographic characteristics, including age and education years, was found (**Supplementary Table 1**). With regard to lifestyles, it was evident that hours of TV viewing among the participants were around 2.28 h on average (2.47 h for men and 2.17 h for women, *P* = 0.028). In surveyed areas, women were more likely to undertake farming than men (65.43% vs. 60.66, *P* = 0.036). Compared to women, more men were often engaged in physical activity (19.90% for men and 14.83% for women, *P* < 0.001). Men also consumed fat more frequently in our study than women (33.46% vs. 11.69%, *P* < 0.001). Further, there was no discrepancy of sleep time between men and women (7.24 vs. 7.18 h, *P* = 0.468). The sociodemographic characteristics and lifestyles of participants across different quantiles of BMI by gender are given in **Table 1**.

**Table 1:** The characteristics of enrolled adult participants across different cutoffs of BMI by gender^a^.

Covariates^b^	Male				Female			
	Underweight	Normal	Overweight	Obesity	Underweight	Normal	Overweight	Obesity
	(q < 5.9)^c^	(5.9 ≤ q < 65.6)	(65.6 ≤ q < 94.0)	(q ≥ 94.0)	(q < 5.9)	(5.8 ≤ q < 66.6)	(66.6 ≤ q < 94.2)	(q ≥ 94.2)
**Number of participants**	62 (5.92)	630 (60.11)	294 (28.05)	62 (5.92)	117 (5.94)	1202 (61.05)	540 (27.43)	110 (5.59)
**BMI (kg/m^2^)**	17.66 (0.65)	21.37 (1.49)	25.57 (1.01)	31.94 (18.07)	17.66 (0.71)	21.59 (1.45)	25.53 (1.11)	30.14 (4.66)
**Sleep duration (hours)**	7.34 (1.93)	7.26 (1.83)	7.20 (1.53)	7.11 (1.54)	7.11 (1.95)	7.21 (2.00)	7.18 (2.03)	7.02 (2.03)
**Age (years)**	53.55 (15.82)	51.33 (12.08)	50.62 (10.82)	49.08 (12.80)	49.29 (14.40)*	48.71 (11.59)	50.46 (10.01)	53.31 (10.75)
**Education level (years)**	7.10 (3.68)*	7.54 (3.29)	8.53 (3.13)	7.81 (2.90)	5.32 (4.01)*	6.39 (3.78)	6.16 (3.85)	5.50 (3.76)
**Hours of TV viewing**	1.87 (1.33)	2.46 (4.16)	2.53 (1.54)	2.80 (1.97)	1.92 (1.33)	2.13 (3.16)	2.16 (1.48)	3.03 (9.76)
**Marital status**								
Unmarried, divorced, widowed	17 (27.4)**	54 (8.6)	15 (5.1)	3 (4.8)	17 (27.4)	54 (8.6)	15 (5.1)	3 (4.8)
Married	45 (72.6)	571 (91.4)	278 (94.9)	59 (95.2)	45 (72.6)	571 (91.4)	278 (94.9)	59(95.2)
**Wealth index**								
Wealthier	19 (30.7)*	214 (34.0)	136 (46.3)	26 (41.9)	19 (30.7)*	214 (34.0)	136 (46.3)	26 (41.9)
Medium	23 (37.1)	230 (36.5)	110 (37.4)	27 (43.6)	23 (37.1)	230 (36.5)	110 (37.4)	27 (43.6)
Poor	20 (32.3)	186 (29.5)	48 (16.3)	9 (14.5)	20 (32.3)	186 (29.5)	48 (16.3)	9 (14.5)
**Farming frequency**								
Often	35 (56.5)**	406 (66.0)	154 (53.3)	28 (45.9)	35 (56.5)**	406 (66.0)	154 (53.3)	28 (45.9)
Sometimes	10 (16.1)	115 (18.7)	66 (22.8)	11 (18.0)	10 (16.1)	115 (18.7)	66 (22.8)	11 (18.0)
None	17 (27.4)	94 (15.3)	69 (23.9)	22 (36.1)	17 (27.4)	94 (15.3)	69 (23.9)	22 (36.1)
**Physical activity**								
Often	18 (29.0)*	122 (19.6)	57 (19.4)	10 (16.1)	18 (29.0)**	122 (19.6)	57 (19.4)	10 (16.1)
Sometimes	1 (1.6)	33 (5.3)	30 (10.2)	7 (11.3)	1 (1.6)	33 (5.3)	30 (10.2)	7 (11.3)
None	43 (69.4)	467 (75.1)	207 (70.4)	45 (72.6)	43 (69.4)	467 (75.1)	207 (70.4)	45 (72.6)
**Frequency of sweets intake**								
Often	30 (48.4)**	312 (50.2)	115 (39.1)	16 (25.8)	30 (48.4)**	312 (50.2)	115 (39.1)	16 (25.8)
Sometimes	25 (40.3)	232 (37.3)	135 (45.9)	32 (51.6)	25 (40.3)	232 (37.3)	135 (45.9)	32 (51.6)
None	7 (11.3)	78 (12.5)	44 (15.0)	14 (22.6)	7 (11.3)	78 (12.5)	44 (15.0)	14 (22.6)
**Frequency of fat intake**								
Often	21 (33.9)**	229 (36.8)	84 (28.6)	14 (22.6)	21 (33.9)	229 (36.8)	84 (28.6)	14 (22.6)
Sometimes	26 (41.9)	308 (49.5)	160 (54.4)	31 (50.0)	26 (41.9)	308 (49.5)	160 (54.4)	31 (50.0)
None	15 (24.2)	85 (13.7)	50 (17.0)	17 (27.4)	15 (24.2)	85 (13.7)	50 (17.0)	17 (27.4)
**Frequency of fried foods intake**								
Often	4 (6.5)	45 (7.3)	22 (7.5)	4 (6.5)	4 (6.5)	45 (7.3)	22 (7.5)	4 (6.5)
Sometimes	43 (69.4)	469 (75.7)	223 (75.9)	51 (82.3)	43 (69.4)	469 (75.7)	223 (75.9)	51 (82.3)
None	15 (24.2)	106 (17.1)	49 (16.7)	7 (11.3)	15 (24.2)	106 (17.1)	49 (16.7)	7 (11.3)
**Smoking** ^d^								
Never smoker	14 (22.6)*	148 (23.8)	81 (27.7)	16 (25.8)	–	–	–	–
Ex-smoker	4 (6.5)	59 (9.5)	47 (16.0)	9 (14.5)	–	–	–	–
Current smoker	44 (71.0)	414 (66.7)	165 (56.3)	37 (59.7)	–	–	–	–
**Alcohol consumption** ^d^								
No	26 (41.9)	209 (33.8)	87 (29.7)	24 (38.7)	–	–	–	–
Yes	36 (58.1)	409 (66.2)	206 (70.3)	38 (61.3)	–	–	–	–

a Data were expressed as numbers or percentages and presented as mean with standard deviation of the mean. All univariate comparisons across subgroups were evaluated using ANOVA, χ^2^, and Kruskal–Wallis as appropriate.

b Missing values: 4 for age, 27 for education years, 31 for sleep time, 117 for TV viewing time, 17 for marriage, 22 for physical activity, 46 for farming, 17 for frequency of sweets intake, 18 for frequency of fat intake, 19 for frequency of fried foods intake, 10 for smoking, 13 for alcohol consumption.

c q denoted quantile of BMI.

d The proportion of smoking and alcohol consumption was too low among women and not given in the table.

*P < 0.05, **P < 0.01 denoted significant difference in the characteristics across different quantiles of BMI.

Moreover, we investigated the differences in sociodemographic characteristics and lifestyles across sleep duration subgroups by gender (**Supplementary Table 2**). Among men, it was clear that the participants in the short and long sleep duration group had a lower education level and greater age. Of them, the economic level was higher in the short and long sleep duration group in comparison with the normal sleep duration group. It was further observed among men that compared to the normal sleep duration group, the frequency of physical activity and proportion of never smoking was higher and the frequency of fried foods intake was lower in the short and long sleep duration group. Among women, the age of the participants and farming frequency were higher and the education years were lower in the normal sleep duration group compared to the other two groups. In the short or long sleep duration group, the overall economic level was lower in contrast to the normal sleep duration group.

### Risk Factors of BMI Among Male Participants in Rural Northwest China

In **Table 2**, the QR and OLS were applied to examine the effects of sociodemographic characteristics and lifestyles on the different quantiles of BMI among men. According to OLS results, the married participants with a high economic level had a greater BMI. Meanwhile, the frequency of sweets intake was negatively associated with BMI among adult men. Similar to OLS results, QR analysis also revealed that the married status and high economic level were also positively related to BMI across the whole quantile of BMI. Regarding their lifestyles, more TV viewing hours every day, frequent fried foods intake, and no smoking were associated with higher BMI among overweight and obese men. Farming frequency and frequent sweets and fat intake were inversely correlated with the BMI across the high quantile of BMI (overweight and obesity).

**Table 2:** The association between sleep duration and the percentiles of BMI among male participants^a,b^.

Covariates	OLS	Underweight		Normal		Overweight		Obesity	
		(BMI <18.5)		(18.5 ≤ BMI < 24.0)		(24.0 ≤ BMI < 28.0)		(BMI ≥28.0)	
		(q <5.9)		(5.9 ≤ q < 66.0)		(66.0 ≤ q < 94.1)		(q ≥ 94.1)	
		q = 2	q = 5	q = 25	q = 50	q = 71	q = 85	q = 95	q = 97
**Sleep duration (hours)**	0.35 (-0.61,1.32)	0.15 (-0.05,0.26)	-0.08 (-0.49,0.33)	-0.21 (-0.71,0.28)	0.48 (-0.17,1.13)	**0.69 (0.13,1.25)**	-0.04 (-0.70,0.62)	0.44 (-0.31,1.19)	**0.77 (0.20,1.35)**
**Squared sleep duration**	-0.03 (-0.10,0.04)	**-0.02 (-0.03,-0.02)**	-0.001 (-0.03,0.03)	0.01 (-0.02,0.05)	-0.04 (-0.08,0.01)	**-0.05 (-0.09,-0.01)**	-0.01 (-0.05,0.05)	-0.02 (-0.08,0.03)	**-0.05 (-0.09,-0.01)**
**Age (years)**	0.01 (-0.03,0.04)	**-0.01 (-0.02,-0.01)**	**-0.02 (-0.03,-0.01)**	0.001 (-0.02,0.02)	**0.03 (0.01,0.05)**	**0.02 (0.01,0.04)**	0.02 (-0.01,0.05)	**0.03 (0.01,0.05)**	0.01 (-0.01,0.03)
**Education level (years)**	-0.02 (-0.15,0.11)	**0.01 (0.01,0.03)**	0.03 (-0.02,0.09)	**0.07 (0.01,0.14)**	0.04 (-0.04,0.13)	0.04 (-0.04,0.11)	-0.01 (-0.10,0.08)	-0.01 (-0.11,0.10)	**-0.08 (-0.16,-0.01)**
**Hours of TV viewing**	-0.01 (-0.11,0.10)	**0.02 (0.01,0.03)**	0.01 (-0.04,0.05)	-0.02 (-0.07,0.03)	-0.01 (-0.07,0.06)	**0.07 (0.02,0.13)**	**0.18 (0.11,0.25)**	**0.21 (0.13,0.28)**	**0.19 (0.13,0.25)**
**Marital status**									
Unmarried, divorced, Widowed	1.00	1.00	1.00	1.00	1.00	1.00	1.00	1.00	1.00
married	**1.66 (0.42,2.90)**	**1.46 (1.32,1.60)**	**1.89 (1.37,2.41)**	**1.79 (1.16,2.42)**	**2.08 (1.25,2.91)**	**1.67 (0.95,2.39)**	**0.89 (0.05,1.74)**	**1.75 (0.79,2.71)**	**2.12 (0.88,2.35)**
**Wealth index**									
Wealthier	**1.39 (0.32,2.47)**	**0.70 (0.58,0.82)**	0.03 (-0.42,0.49)	0.50 (-0.05,1.05)	**0.81 (0.09,1.53)**	**1.14 (0.51,1.76)**	**1.06 (0.33,1.79)**	0.33 (-0.50,1.17)	**1.62 (1.13,2.41)**
Medium	0.68 (-0.29,1.66)	**0.45 (0.34,0.56)**	-0.31 (-0.72,0.10)	0.01 (-0.50,0.50)	**0.71 (0.06,1.37)**	**0.84 (0.28,1.41)**	**0.80 (0.14,1.46)**	**0.98 (0.22,1.73)**	**1.10 (0.20,1.35)**
Poor	1.00	1.00	1.00	1.00	1.00	1.00	1.00	1.00	1.00
**Farming frequency**									
Often	-0.41 (-1.33,0.51)	0.09 (-0.02,0.19)	-0.04 (-0.43,0.35)	-0.41 (-0.88,0.07)	**-0.97 (-1.59,-0.35)**	**-1.17 (-1.71,-0.64)**	**-1.06 (-1.69,-0.43)**	**-1.56 (-2.28,-0.85)**	**-1.04 (-1.58,-0.49)**
Sometimes	-0.40 (-1.49,0.70)	**-0.45 (-0.58,-0.33)**	-0.07 (-0.53,0.39)	**0.06 (-0.50,0.62)**	**-0.43 (-1.17,0.31)**	**-0.34 (-0.97,0.23)**	**-0.60 (-1.35,0.15)**	**-1.40 (-2.25,-0.55)**	-0.61 (-1.26,0.05)
None	1.00	1.00	1.00	1.00	1.00	1.00	1.00	1.00	1.00
**Physical activity**									
Often	-0.52 (-1.43,0.40)	**0.18 (0.08,0.29)**	-0.24 (-0.63,0.14)	-0.20 (-0.67,0.275)	-0.19 (-0.80,0.43)	-0.45 (-0.98,0.08)	**-0.63 (-1.25,-0.01)**	**-0.91 (-1.62,-0.20)**	**-0.96 (-1.50,-0.41)**
Sometimes	0.55 (-0.84,1.94)	**0.57 (0.41,0.73)**	0.34 (-0.25,0.92)	0.21 (-0.50,0.92)	**1.43 (0.49,2.36)**	0.69 (-0.12,1.49)	0.14 (-0.81,1.09)	0.82 (-0.26,1.89)	0.70 (-0.12,1.53)
None	1.00	1.00	1.00	1.00	1.00	1.00	1.00	1.00	1.00
**Frequency of sweets intake**									
Often	**-1.20 (-2.30,-0.11)**	-0.10 (-0.22,0.03)	-0.36 (-0.82,0.10)	**-0.89 (-1.45,-0.34)**	**-1.09 (-1.82,-0.36)**	**-1.42 (-2.05,-0.79)**	**-1.72 (-2.47,-0.98)**	**-2.24 (-3.09,-1.40)**	**-1.76 (-2.41,-1.11)**
Sometimes	-0.06 (-1.14,1.03)	0.01 (-0.12,0.13)	-0.37 (-0.82,0.09)	-0.35 (-0.90,0.21)	-0.28 (-1.02,0.45)	-0.30 (-0.93,0.33)	-0.36 (-1.10,0.38)	**-1.06 (-1.91,-0.22)**	-0.51 (-1.15,0.14)
None	1.00	1.00	1.00	1.00	1.00	1.00	1.00	1.00	1.00
**Frequency of fat intake**									
Often	-1.20 (-1.67,0.52)	**0.23 (0.10,0.35)**	**0.57 (0.11,1.03)**	-0.23 (-0.79,0.34)	-0.34 (-1.08,0.40)	-0.55 (-1.19,0.09)	**-1.15 (-1.90,-0.40)**	**-1.07 (-1.92,-0.21)**	-0.09 (-0.74,0.56)
Sometimes	-0.06 (-0.86,1.16)	**0.39 (0.27,0.45)**	**0.71 (0.28,1.13)**	0.35 (-0.17,0.86)	0.17 (-0.51,0.85)	-0.18 (-0.76,0.41)	**-0.82 (-1.51,-0.14)**	**-1.14 (-1.93,-0.36)**	**-0.71 (-1.31,-0.11)**
None	1.00	1.00	1.00	1.00	1.00	1.00	1.00	1.00	1.00
**Frequency of fried foods intake**									
Often	0.81 (-0.76,2.39)	0.02 (-0.16,0.20)	0.46 (-0.20,1.12)	0.69 (-0.11,1.50)	0.55 (-0.51,1.61)	0.63 (-0.28,1.54)	0.57 (-0.45,1.64)	0.47 (-0.75,1.69)	0.64 (-0.29,1.58)
Sometimes	0.53 (-0.43,1.50)	**0.26 (0.15,0.37)**	**0.45 (0.05,0.86)**	-0.14 (-0.64,0.35)	0.24 (-0.40,0.89)	0.34 (-0.22,0.90)	0.30 (-0.36,0.96)	**1.53 (0.78,2.27)**	**1.64 (1.07,2.21)**
None	1.00	1.00	1.00	1.00	1.00	1.00	1.00	1.00	1.00
**Smoking**									
Never smoker	0.30 (-0.54,1.14)	**0.64 (0.55,0.74)**	**0.55 (0.20,0.90)**	**0.53 (0.10,0.96)**	0.35 (-0.22,0.91)	**0.49 (0.01,0.98)**	0.27 (-0.30,0.84)	**1.02 (0.37,1.67)**	**0.85 (0.35,1.35)**
Ex-smoker	0.63 (-0.51,1.78)	**0.78 (0.65,0.91)**	**0.79 (0.31,1.27)**	**0.98 (0.39,1.57)**	**1.17 (0.40,1.94)**	**0.77 (0.11,1.44)**	0.65 (-0.13,1.43)	-0.17 (-1.06,0.72)	-0.29 (-0.98,0.39)
Current smoker	1.00	1.00	1.00	1.00	1.00	1.00	1.00	1.00	1.00
**Alcohol consumption**									
No	1.00	1.00	1.00	1.00	1.00	1.00	1.00	1.00	1.00
Yes	0.26 (-0.49,1.02)	-0.03 (-0.11,0.06)	0.21 (-0.11,0.53)	0.36 (-0.02,0.75)	0.14(-0.36,0.65)	0.15(-0.29,0.59)	0.10 (-0.42,0.61)	-0.39 (-0.97,0.20)	**-0.52 (-0.97,-0.07)**

a Values were β-estimates (95% CI) of covariates on the percentiles of BMI in the table; coefficients significant at the 5% level were bold. q denoted the percentiles of BMI.

b The association between continuous sleep time and the percentiles of BMI was assessed using OLS regression and quantile regression model.

### Risk Factors of BMI Among Female Participants in Rural Northwest China

Among women, the positive association of BMI with wealth index and hours of TV viewing could be identified using OLS regression (**Table 3**). Based on QR, results indicated that BMI across the whole quantiles was progressively rising, when female participants’ age, hours of TV viewing, and household economic level were increasing. We also observed a positive relationship between frequent fat and fried foods intake and BMI among obese female participants. Women with a higher education level and frequency of sweets intake and farming were more likely to have a lower BMI across higher quantiles of BMI.

**Table 3:** The association between sleep duration and the percentiles of BMI among female participants^a,b^.

Covariates	OLS	Underweight		Normal		Overweight		Obesity	
		(BMI < 18.5)		(18.5 ≤ BMI < 24.0)		(24.0 ≤ BMI < 28.0)		(BMI≥28.0)	
		(q < 5.9)		(5.9 ≤ q < 67.0)		(67.0 ≤ q < 94.4)		(q ≥ 94.4)	
		q = 2	q = 5	q = 15	q = 50	q = 68	q = 80	q = 97	q = 99
**Sleep duration (hours)**	-0.15 (-0.51,0.21)	**-0.64 (-0.94,-0.34)**	**-0.48 (0.83,-0.12)**	-0.35 (-0.76,0.06)	-0.11 (-0.53,0.31)	-0.29 (-0.71,0.11)	0.07 (-0.40,0.55)	-0.34 (-0.99,0.30)	-0.11 (-0.35,0.13)
**Squared sleep duration**	0.01 (-0.02,0.04)	**0.04 (0.02,0.06)**	**0.03 (0.01,0.06)**	**0.02 (0.01,0.05)**	0.01 (-0.02,0.04)	0.02 (-0.01,0.05)	-0.01 (-0.04,0.03)	0.02 (-0.03,0.07)	0.01 (-0.01,0.02)
**Age (years)**	**0.04 (0.02,0.05)**	**0.02 (0.01,0.04)**	**0.02 (0.01,0.04)**	**0.03 (0.01,0.05)**	**0.04 (0.02,0.06)**	**0.04 (0.02,0.05)**	**0.05 (0.03,0.07)**	**0.04 (0.01,0.07)**	**0.04 (0.03,0.05)**
**Education level (years)**	-0.02 (-0.07,0.03)	**0.08 (0.04,0.12)**	**0.07 (0.03,0.12)**	0.045 (-0.01,0.10)	0.02 (-0.04,0.08)	-0.07 (-0.11,0.01)	**-0.09 (-0.16,-0.03)**	-0.07 (-0.16,0.02)	**-0.29 (-0.32,-0.25)**
**Hours of TV viewing**	**0.06 (0.01,0.10)**	**0.06 (0.03,0.10)**	**0.05 (0.01,0.09)**	0.04 (-0.01,0.08)	**0.06 (0.01,0.11)**	**0.09 (0.04,0.14)**	**0.16 (0.10,0.21)**	**0.12 (0.05,0.19)**	**0.34 (0.31,0.35)**
**Marital status**									
Unmarried, divorced, Widowed	1.00	1.00	1.00	1.00	1.00	1.00	1.00	1.00	1.00
married	0.34 (-0.22,0.94)	**-0.71 (-1.18,-0.23)**	-0.44 (-1.01,0.12)	0.30 (-0.35,0.95)	0.53 (-0.14,1.20)	0.34 (-0.32,1.01)	-0.23 (-0.99,0.52)	-0.54 (-1.56,0.50)	**0.70 (0.32,1.09)**
**Wealth index**									
Wealthier	**0.66 (0.20,1.12)**	0.18 (-0.20,0.56)	0.29 (-0.16,0.74)	**0.70 (0.19,1.23)**	**0.56 (0.03,1.10)**	**0.74 (0.21,1.27)**	**0.79 (0.19,1.3)**	0.21 (-0.61,1.03)	**0.98 (0.67,1.28)**
Medium	**0.50 (0.12,0.87)**	**-0.03 (-0.34,0.29)**	0.21 (-0.16,0.58)	0.18 (-0.25,0.60)	0.31 (-0.13,0.74)	**0.64 (0.20,1.07)**	**0.62 (0.13,1.12)**	**0.82 (0.15,1.49)**	**2.50 (2.25,2.75)**
Poor	1.00	1.00	1.00	1.00	1.00	1.00	1.00	1.00	1.00
**Farming frequency**									
Often	0.05 (-0.36,0.47)	**1.09 (0.75,1.43)**	**0.84 (0.44,1.24)**	**0.58 (0.12,1.04)**	-0.14 (-0.61,0.34)	-0.05 (-0.52,0.42)	-0.16 (-0.70,0.38)	**-0.21 (-0.94,0.52)**	**-1.11 (-1.38,-0.84)**
Sometimes	0.36 (-0.14,0.85)	**1.73 (1.32,2.13)**	**1.29 (0.81,1.77)**	**0.97 (0.41,1.53)**	0.25 (-0.32,0.83)	-0.05 (-0.61,0.52)	-0.08 (-0.73,0.57)	**-0.29 (-1.17,0.59)**	**-1.88 (-2.21,-1.55)**
None	1.00	1.00	1.00	1.00	1.00	1.00	1.00	1.00	1.00
**Physical activity**									
Often	0.29 (-0.13,0.72)	0.33 (-0.02,0.68)	0.12 (-0.29,0.54)	0.31 (-0.17,0.80)	0.27 (-0.23,0.77)	**0.50 (0.01,0.99)**	0.16 (-0.40,0.72)	0.67 (-0.09,1.43)	**1.24 (0.96,1.52)**
Sometimes	0.15 (-0.54,0.84)	0.43 (-0.14,1.00)	0.21 (-0.46,0.88)	0.20 (-0.58,0.98)	-0.04 (-0.84,0.76)	0.45 (-0.34,1.24)	0.56 (-0.34,1.64)	0.04 (-1.18,1.27)	**-1.38 (-1.84,-0.93)**
None	1.00	1.00	1.00	1.00	1.00	1.00	1.00	1.00	1.00
**Frequency of sweets intake**									
Often	**-1.00 (-1.51,-0.50)**	-0.07 (-0.49,0.35)	-0.15 (-0.65,0.34)	-0.38 (-0.96,0.19)	**-1.09 (-1.68,-0.50)**	**-1.18 (-1.77,-0.60)**	**-2.08 (-2.74,-1.41)**	**-0.93 (-1.83,-0.03)**	**-0.58 (-0.91,-0.24)**
Sometimes	-0.32 (-0.82,0.19)	**0.60 (0.19,1.02)**	**0.73 (0.24,1.22)**	0.436 (-0.13,1.00)	-0.52 (-1.10,0.07)	**-0.59 (-1.17,-0.01)**	**-1.55 (-2.20,-0.89)**	0.30 (-0.60,1.19)	-0.19 (-0.52,0.14)
None	1.00	1.00	1.00	1.00	1.00	1.00	1.00	1.00	1.00
**Frequency of fat intake**									
Often	-0.23 (-0.74,0.27)	-0.40 (-0.81,0.02)	**-0.56 (-1.05,-0.07)**	-0.136 (-0.71,0.43)	-0.08 (-0.66,0.51)	-1.18 (-0.38,0.78)	-0.33 (-0.99,0.33)	-0.43 (-1.33,0.46)	**1.80 (1.47,2.14)**
Sometimes	-0.18 (-0.50,0.15)	-0.25 (-0.52,0.02)	-0.25 (-0.56,0.07)	-0.15 (-0.52,0.21)	-0.16 (-0.53,0.21)	-0.59 (-0.35,0.39)	-0.18 (-0.60,0.24)	-0.22 (-0.79,0.35)	-0.14 (-0.35,0.08)
None	1.00	1.00	1.00	1.00	1.00	1.00	1.00	1.00	1.00
**Frequency of fried foods intake**									
Often	0.29 (-0.39,0.96)	0.20 (-0.36,0.76)	0.26 (-0.40,0.92)	0.19 (-0.57,0.95)	0.29 (-0.50,1.07)	0.09 (-0.68,0.87)	0.56 (-0.32,1.44)	-0.09 (-1.29,1.11)	**0.55 (0.10,0.99)**
Sometimes	0.29 (-0.10,0.67)	0.07 (-0.25,0.39)	0.16 (-0.21,0.53)	0.17 (-0.27,0.60)	0.02 (-0.42,0.47)	0.24 (-0.20,0.69)	0.32 (-0.18,0.82)	**0.73 (0.04,1.41)**	**2.21 (1.95,2.46)**
None	1.00	1.00	1.00	1.00	1.00	1.00	1.00	1.00	1.00

a Values were β-estimates (95% CI) of covariates on the percentiles of BMI in the table; coefficients significant at the 5% level were bold. q denoted the percentiles of BMI.

b The association between continuous sleep time and the percentiles of BMI was assessed using OLS regression and quantile regression model.

### The Association of Sleep Duration With BMI in Rural Northwest China

**Figure 1:**
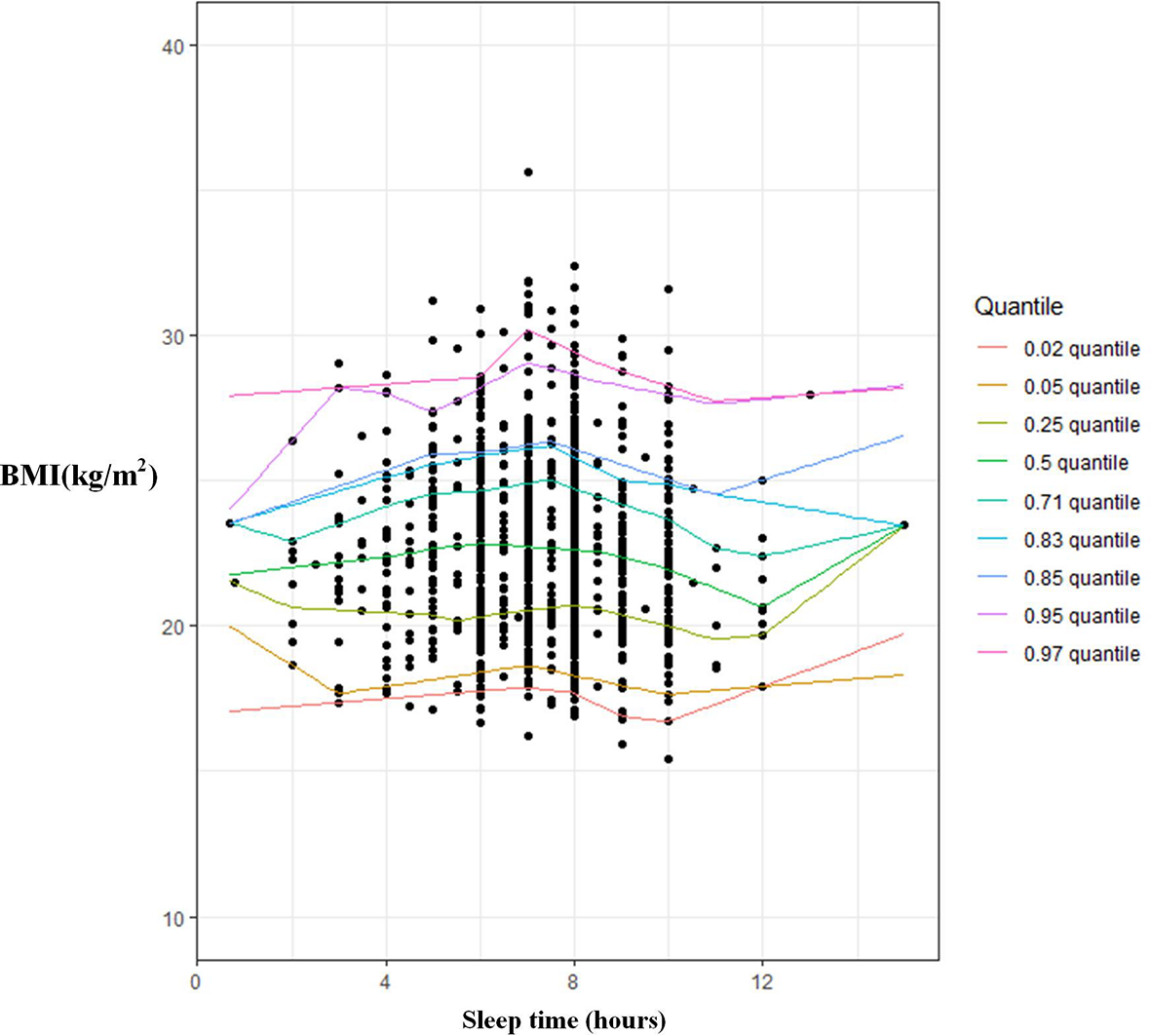
The association between sleep duration and BMI among men using multivariate quantitle regression model.

Adjusting for confounding factors including sociodemographic characteristics and lifestyles using quantile regressions, it was clear in men there is an inverse U-shaped relationship between sleep time and BMI beyond the 66th percentile (BMI ≥ 24) (**Table 2**, **Figure 1**). That is, the coefficients of sleep time were positive, while the coefficient of SleepSQ was negative. Although a similar relationship between sleep time and BMI was also observed in men using OLS regression, it was not statistically significant (**Table 2**).

**Figure 2:**
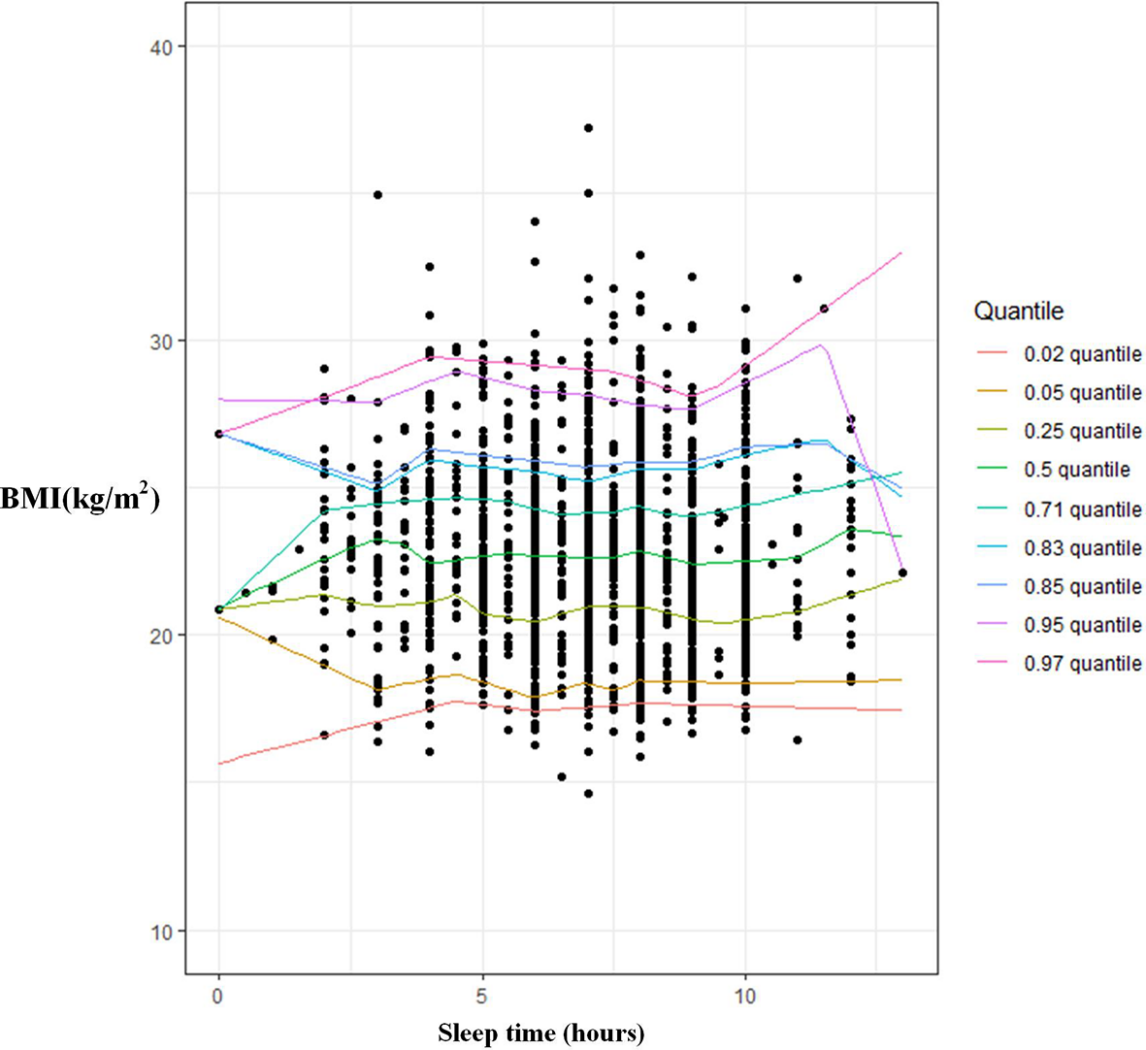
The association between sleep duration and BMI among women using multivariate quantitle regression model.

A U-shaped relationship with sleep duration among women using OLS regression was found, but the coefficients of sleep duration and squared sleep duration did not reach statistical significance (**Table 3**). However, quantile regression presented a significant U-shaped relationship between BMI and sleep duration, as sleep duration had a negative coefficient, while SleepSQ had a positive coefficient (**Table 3**, **Figure 2**).

**Figure 3:**
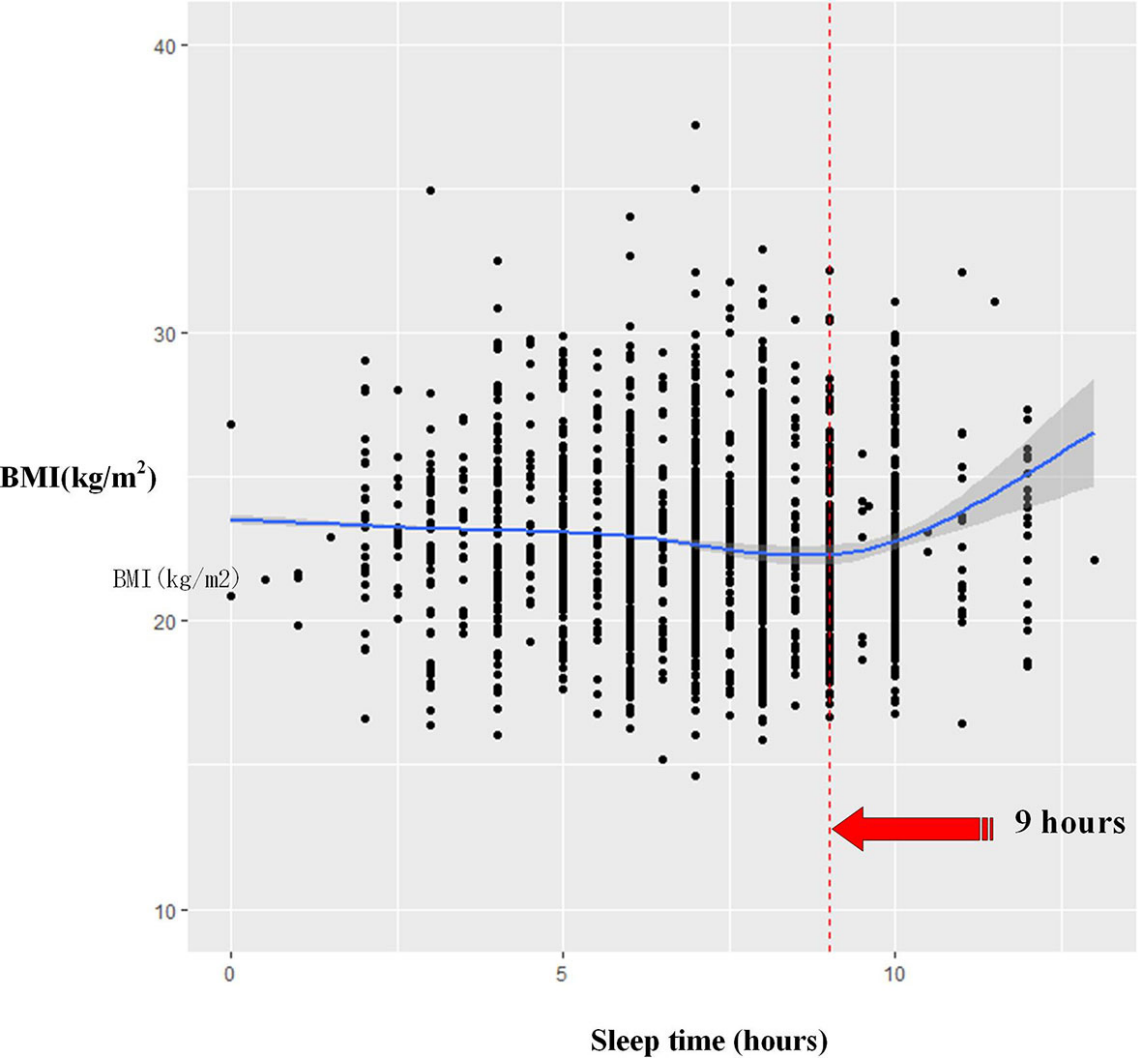
The association between sleep duration and BMI among women using restricted cubic splines.

**Figure 4:**
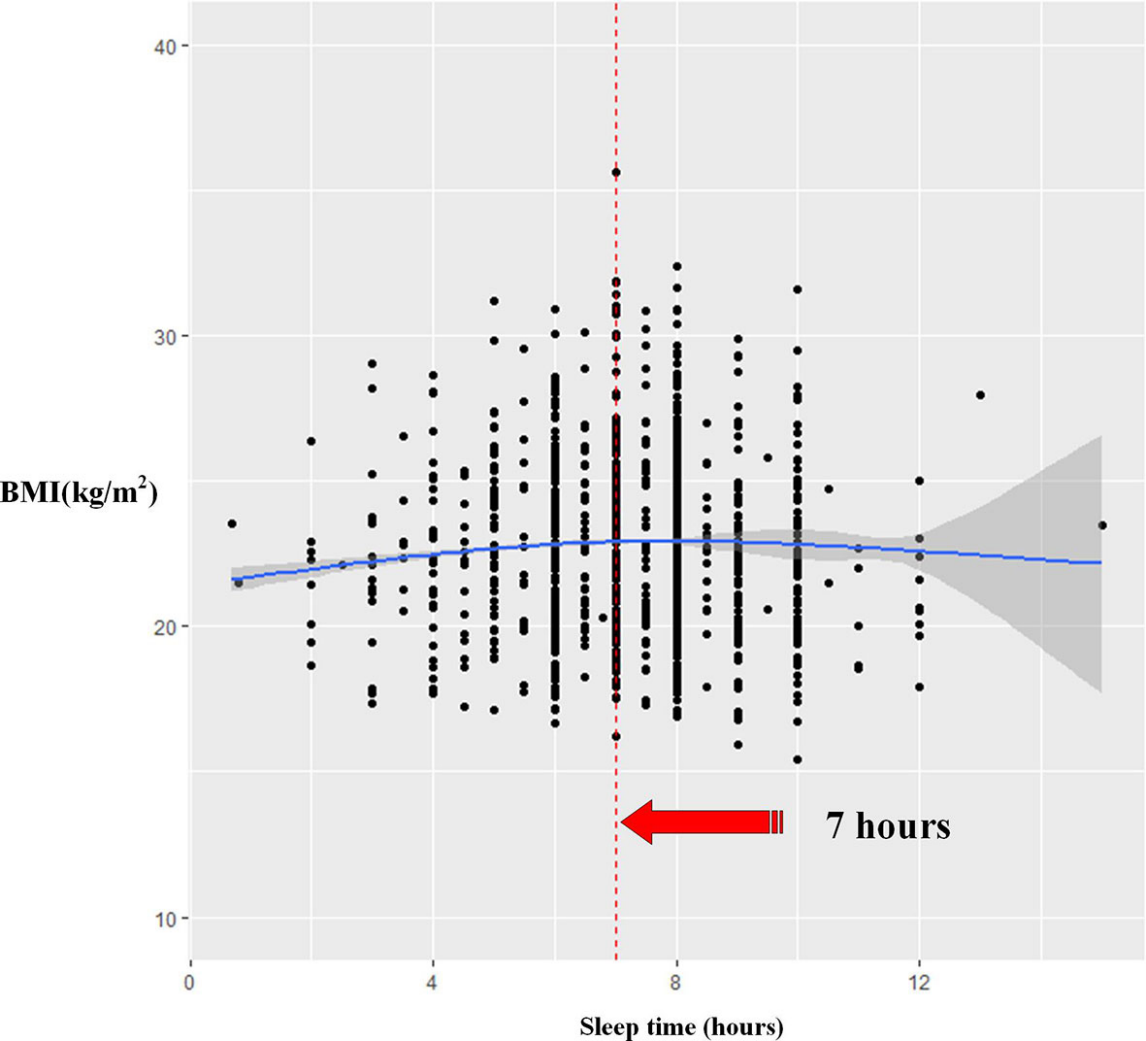
The association between sleep duration and BMI among men using restricted cubic splines.

The restricted cubic splines were further adopted to explore the relationship between sleep duration and BMI by gender (**Figures 3** and **4**). After adjustment for possible confounders in women, a U-shaped relationship was observed in participants, who had the lowest BMI at approximately 9 h of sleep duration. An inverse U-shaped relationship was found in men; namely, when sleep duration approached approximately 8 h, the BMI would be the highest. The results were almost unchanged when we removed underweight participants (**Supplementary Tables 3, 4**).

## Discussion

In this study, we examined the association between sleep duration and BMI by gender and explored the possible risk factors of BMI among adult residents in rural Hanzhong, Northwest China, using OLS and quantile regressions, respectively. We also used restricted cubic splines to further determine the relationship between BMI and sleep duration by gender. Our results indicated that there was a significant difference in the sleep duration–BMI association by gender in rural Northwest China.

Our findings clearly showed the presence of a U-shaped relationship between sleep duration and BMI in women using quantile regression. Both short and long sleep durations were related to a higher BMI among female participants; those who slept for approximately 9 h have the lowest BMI. Several studies had also revealed a similar association between sleep duration and BMI in women. One study recruiting more than 40,834 middle-aged Australian adults had indicated that short sleep (<7-h sleep) was associated with obesity in women, whereas long sleep (≥9-h sleep) was not associated with obesity (24). Chaput et al. by means of a meta-analysis confirmed that when compared with women who slept for 7 to 8 hours per day, the risk for overweight/obesity was 1.38 for those with 9 to 10 h of sleep and 1.69 for those with 5 to 6 h of sleep (25). Itani et al. showed that short sleep duration (<6 h/day) was associated with a 36% absolute increase in the incidence of obesity among women, while long sleep duration (>9 h/day) was associated with a 12% absolute increase in the incidence of obesity compared with normal sleep duration (13, 14). Cho et al. also found that sleep duration ≤ 5 h/day was likely to increase obesity in women 30–49 years of age, compared with sleep duration of 6 to 8 h/day (18). This relationship among women in our study seems to be consistent with previous studies and persists after adopting the restricted cubic spline model.

Although the study confirms a U-shaped association between sleep duration and BMI among women, the mechanisms do not seem straightforward. Some studies have indicated that short sleep duration was associated with decreased leptin and increased ghrelin, which had also been observed to be associated with increased appetite (26, 27). These hormone alterations may contribute to the BMI increase that occurs with sleep deprivation (27, 28). In animal studies indicating a similar relationship between sleep and metabolism, furthermore, prolonged sleep deprivation increased food intake and energy expenditure in rats (15). Studies suggested that long sleepers may have low energy expenditure or may have poor sleep quality because of the long time in bed (29). In this study, however, we did not observe any difference in food intakes in women between short or long and normal sleep duration.

Among women, moreover, female BMI was positively associated with age and household economic level, but the negative relationship between BMI and education year and farming frequency was also observed, which were consistent with our previous study (12). When compared to sleep duration of 7–9 h, it was clear that the age of participants was higher and their education year was lower in the short sleep duration group. This could partly explain the higher risk for BMI in the short sleep duration group, suggesting the importance of age and education factors. Additionally, the household economic level was increasing and farming frequency was decreasing with sleep duration among women (see **Supplementary Table 2**). The characteristics of household economic status and farming frequency appeared to contribute to the positive association between long sleep and BMI among women.

This result among men was somewhat unexpected compared to the women’s result and showed that in overweight and obese men the coefficient of sleep duration was positive, while the coefficient of SleepSQ was negative. Thus, an inverse U-shaped relationship between sleep duration and BMI was likely to exist among men in rural Hanzhong of Northwest China, and men with short or long sleep duration might have a lower BMI. According to the restricted cubic spline model, the male participants who slept for approximately 7 h had the highest BMI. The meta-analysis by Itani et al. indicated a U-shaped relationship between sleep duration and BMI among men (13, 14). A study in Korean adults found that for elderly individuals (≥65 years), there was a negative association between sleep duration ≤5 h/day and obesity in men (18). Previous studies have reported inconsistent results when examining the association between sleep duration and obesity in men.

QR analysis revealed that men’s economic status was positively correlated with BMI across the whole quantile of BMI. Less physical activities were associated with a higher BMI among overweight men. Across the three sleep duration subgroups among men, it was clear that the economic level was lower in short and long sleep duration than that in normal sleep duration. In addition, the intake of fried foods which was more likely to increase the BMI of men was higher among men with 7–9 h of sleep duration compared to those with short and long sleep duration. It is likely that the pattern above supports the hypothesis of the concave relationship in men.

To date, however, there is lack of exact explanations for gender differences in the relationship between sleep duration and obesity. The first possible reason is that men and women are exposed to different types of stress, including socioeconomic and cultural factors. In our study, the household economic level of women increased with sleep duration and was the highest in the long sleep duration group, whereas the economic level of men was lower in the short and long sleep duration group. In order to bring in more money and supplement farming income, most of the local male residents leave the village to work temporary jobs in urban areas, while they occupy themselves with farming in rural areas. Therefore, short or long sleepers among men perform more vigorous physical activities to spend more energy than the average sleepers in study areas (25). In addition, restriction of sleep among women would lead to increased daytime sleepiness, which may hinder participation in farming activity and preparation of healthy food, as opposed to purchasing pre-prepared and often less nutritionally optimal food options (16, 30). These might in part contribute to the gender-specific pattern in the relationship between sleep duration and BMI.

Another explanation for the inconsistent results by gender may be a wide age range in the study. Previous studies have shown a negative association between sleep duration ≤5 h/day and obesity in the ≥65-year-old male group, with a prominent positive relationship between sleep duration ≤5 h/day and obesity in women at 30–49 years of age (18). After examining the ages of participants in our studies, varying age ranges, such as 18 to 80 years, and the higher proportion of participants ≥65 years in men (10.8%) than in women (7.3%), to some extent may explain the gender-specific results. Apart from aforementioned explanations, the different standards of normal sleep hours and self-reported sleep duration are also likely reasons for the gender discrepancy in the relationship between sleep duration and obesity in our study. Further epidemiological studies will be needed to investigate the gender difference and examine specific mechanisms.

It is common knowledge that the coefficient in the OLS model only reflect the central tendency of the marginal effects of sleep duration on the conditional mean of the BMI. However, our results highlighted the importance of examining the effects of sleep duration across the entire distribution of BMI. On the basis of OLS regression, we did not find any statistically significant correlation between sleep time and BMI, while there was a clear effect of sleep duration on the different percentiles of BMI using quantile regression controlling for other confounders including sociodemographic characteristics and lifestyles of participants. In past studies, similarly, quantile regression was also able to reveal significant relationships between various covariates and BMI in certain quantiles when OLS models showed insignificant results (31, 32). Thus, the utilization of quantile regression was the largest strength in the study, which may provide a clear visual depiction of the sleep duration–BMI relationship among residents by gender in rural Hanzhong of Northwest China.

In our study, several limitations should be noted in the explanation of the results. Due to the cross-sectional study, firstly, any cause–effect conclusions could not be drawn. Secondly, although our results suggested a relation between sleep duration and BMI by gender, the observed associations might be subject to unmeasured confounders. Thirdly, information on sleep duration was collected by self-report. Therefore, we could not rule out the possibility of information bias. Fourthly, as the study was confined to rural Northwest China, the conclusions from this study might not be generalizable to other areas in China. To our knowledge, however, this is the first study to investigate the relationship between sleep duration and BMI by gender using quantile regression in rural areas of Northwest China, which might fill the gap and have important implications for obesity prevention and control.

## Conclusions

In summary, quantile regression could provide a clear visual picture of the relationship between sleep duration and BMI in adults. After controlling for sociodemographic characteristics and lifestyles, a U-shaped relationship between sleep duration and BMI had been found in women; oppositely, an inverse U-shaped relationship between sleep duration and BMI was observed in men in rural Hanzhong of Northwest China. The women who sleep for approximately 9 h had the lowest BMI, and when sleep duration approached approximately 7 h among men, their BMI would be the highest.

## Data Availability Statement

The original contributions presented in the study are included in the article/**Supplementary Material**. Further inquiries can be directed to the corresponding author.

## Ethics Statement

The study was conducted according to the guidelines of the Declaration of Helsinki and approved by the Human Research Ethics Committee of the Xi’an Jiaotong University Health Science Center (Number: 2002001). The patients/participants provided their written informed consent to participate in this study.

## Author Contributions

LR and LP designed the study. LR, LC, and YK conducted the statistical analysis. YZ, FC, and YK collected the data. LR and LP drafted the manuscript. LP made the critical revisions. All authors contributed to the article and approved the submitted version.

## Funding

All the work was supported by the National Natural Science Foundation of China (72174167, 81602928) and the Natural Science Foundation of Shaanxi Province (2021JM-031).

## Conflict of Interest

The authors declare that the research was conducted in the absence of any commercial or financial relationships that could be construed as a potential conflict of interest.

## Publisher’s Note

All claims expressed in this article are solely those of the authors and do not necessarily represent those of their affiliated organizations, or those of the publisher, the editors and the reviewers. Any product that may be evaluated in this article, or claim that may be made by its manufacturer, is not guaranteed or endorsed by the publisher.

## Abbreviations

OLS, ordinary least square; QR, quantile regression; BMI, body mass index; WGOC, Working Group on Obesity in China; SleepSQ, squared sleep duration.
