# Supplementary material for: Gender-Specific Association Between Sleep Duration and Body Mass Index in Rural China
Source: Front Endocrinol (Lausanne). 2022 May 31;13:877100. doi: 10.3389/fendo.2022.877100 (PMC9193222; doi:10.3389/fendo.2022.877100)
Supplement: Supplementary file 1 [file DataSheet_1.docx]

Supplementary Table 1 Baseline characteristics of enrolled adult participants by gender ^a^

|  | Total |  | Male |  | Female |  | *P* |
| --- | --- | --- | --- | --- | --- | --- | --- |
| *Quantitative variables* | $\bar{x}$(s) |  | $\bar{x}$(s) |  | $\bar{x}$(s) |  |  |
| **BMI** | 22.86(3.04) |  | 22.81(3.07) |  | 22.89(3.02) |  | 0.502 |
| **Age(years)** | 50.05(11.65) |  | 51.13(12.06) |  | 49.48(11.38) |  | <0.001 |
| **Education level(years)** | 6.77(3.72) |  | 7.81(3.28) |  | 6.21(3.82) |  | <0.001 |
| **Sleep time(hours)** | 7.20(1.92) |  | 7.24(1.74) |  | 7.18(2.00) |  | 0.468 |
| **Hours of TV viewing** | 2.28(3.44) |  | 2.47(3.37) |  | 2.17(3.47) |  | 0.028 |
| *Categorical variables* |  |  |  |  |  |  |  |
| **Marital status** |  |  |  |  |  |  | 0.551 |
| Unmarried, Divorced, Widowed | 244(8.13) |  | 89(8.54) |  | 155(7.92) |  |  |
| Married | 2756(91.87) |  | 953(91.46) |  | 1803(92.08) |  |  |
| **Wealth index** |  |  |  |  |  |  | <0.001 |
| Wealthier | 905(30.01) |  | 395(37.69) |  | 510(25.90) |  |  |
| Medium | 1206(39.97) |  | 390(37.21) |  | 816(41.44) |  |  |
| Poor | 906(30.02) |  | 263(25.10) |  | 643(32.66) |  |  |
| **Farming frequency** |  |  |  |  |  |  | 0.036 |
| Often | 1895(63.78) |  | 623(60.66) |  | 1272(65.43) |  |  |
| Sometimes | 539(18.15) |  | 202(19.67) |  | 337(17.34) |  |  |
| None | 537(18.07) |  | 202(19.67) |  | 335(17.23) |  |  |
| **Physical activity** |  |  |  |  |  |  | <0.001 |
| Often | 497(16.59) |  | 207(19.90) |  | 290(14.83) |  |  |
| Sometimes | 163(5.45) |  | 71(6.83) |  | 92(4.71) |  |  |
| None | 2335(77.96) |  | 762(73.27) |  | 1573(80.46) |  |  |
| **Frequency of sweets intake** |  |  |  |  |  |  | 0.031 |
| Often | 1349(44.97) |  | 473(45.48) |  | 876(44.69) |  |  |
| Sometimes | 1295(43.17) |  | 424(40.77) |  | 871(44.43) |  |  |
| None | 356(11.86) |  | 143(13.75) |  | 213(10.88) |  |  |
| **Frequency of fat intake** |  |  |  |  |  |  | <0.001 |
| Often | 577(19.24) |  | 348(33.46) |  | 229(11.69) |  |  |
| Sometimes | 1566(52.22) |  | 525(50.48) |  | 1041(53.14) |  |  |
| None | 856(28.54) |  | 167(16.06) |  | 689(35.17) |  |  |
| **Frequency of fried foods intake** |  |  |  |  |  |  | 0.154 |
| Often | 208(6.94) |  | 75(7.23) |  | 133(6.79) |  |  |
| Sometimes | 2222(74.11) |  | 786(75.72) |  | 1436(73.26) |  |  |
| None | 568(18.95) |  | 177(17.05) |  | 391(19.95) |  |  |

^a^ Data was expressed as numbers or percentages and presented as mean with standard deviation of the mean. All univariate comparisons across subgroups were evaluated using ANOVA, χ^2^ and Kruskal-Wallis as appropriate.

Supplementary Table 2 Baseline characteristics of enrolled adult participants across different cutoffs of sleep time by gender ^a^

| Covariates | Male | | |  | Female | | |
| --- | --- | --- | --- | --- | --- | --- | --- |
|  | <7 hours | 7-9 hours | >9 hours |  | <7 hours | 7-9 hours | >9 hours |
| *Quantitative variables* | $\bar{x}$(s) | $\bar{x}$(s) | $\bar{x}$(s) |  | $\bar{x}$(s) | $\bar{x}$(s) | $\bar{x}$(s) |
| **BMI** | 22.77(2.96) | 22.94(6.81) | 22.16(3.06) |  | 22.94(3.16) | 22.92(3.28) | 22.96(3.11) |
| **Age(years)** | 53.36(10.72)** | 49.95(12.01) | 50.78(15.36)** |  | 53.62(10.04)** | 47.40(11.37) | 47.43(11.91) |
| **Education level(years)** | 7.48(3.21)** | 8.20(3.18) | 6.53(3.71)** |  | 5.19(3.95)** | 6.80(3.61) | 6.36(3.79) |
| **Hours of TV viewing** | 2.39(1.50) | 2.55(4.22) | 2.26(1.68) |  | 1.98(1.60) | 2.28(4.41) | 2.10(1.37) |
| *Categorical variables* | n (%) | n (%) | n (%) |  | n (%) | n (%) | n (%) |
| **Marital status** |  |  |  |  |  |  |  |
| Unmarried, Divorced, Widowed | 24(7.14) | 53(8.76) | 12(12.50) |  | 61(9.56)* | 73(6.69) | 22(10.33)* |
| Married | 312(92.86) | 552(91.24) | 84(87.50) |  | 577(90.44) | 1018(93.31) | 191(89.67) |
| **Wealth index** |  |  |  |  |  |  |  |
| Wealthier | 108(31.95)** | 256(42.31) | 29(30.21)** |  | 131(20.44)** | 309(28.24) | 67(31.31)** |
| Medium | 127(37.57) | 223(36.86) | 36(37.50) |  | 239(37.29) | 487(44.52) | 82(38.32) |
| Poor | 103(30.47) | 126(20.83) | 31(32.29) |  | 271(42.28) | 298(27.24) | 65(30.37) |
| **Farming frequency** |  |  |  |  |  |  |  |
| Often | 210(62.50) | 352(59.26) | 61(64.21) |  | 429(67.45)* | 708(65.13) | 127(59.91)* |
| Sometimes | 59(17.56) | 124(20.88) | 19(20.00) |  | 93(14.62) | 207(19.04) | 36(16.98) |
| None | 67(19.94) | 19(19.87) | 15(15.79) |  | 114(17.92) | 172(15.82) | 49(23.11) |
| **Physical activity** |  |  |  |  |  |  |  |
| Often | 78(23.08)* | 103(17.02) | 24(25.26)* |  | 100(15.63) | 167(11.92) | 22(15.74) |
| Sometimes | 16(4.73) | 51(8.43) | 4(4.21) |  | 23(3.59) | 57(5.22) | 11(5.14) |
| None | 244(72.19) | 451(74.55) | 67(70.53) |  | 517(80.78) | 356(79.47) | 692(84.58) |
| **Frequency of sweets intake** |  |  |  |  |  |  |  |
| Often | 160(47.34) | 270(44.70) | 43(44.79) |  | 285(44.46) | 481(44.01) | 108(50.47) |
| Sometimes | 137(40.53) | 252(41.72) | 35(42.45) |  | 281(43.84) | 500(45.75) | 83(38.79) |
| None | 41(12.13) | 82(13.58) | 18(18.75) |  | 75(11.70) | 112(10.25) | 23(10.75) |
| **Frequency of fat intake** |  |  |  |  |  |  |  |
| Often | 129(38.17)* | 195(32.28) | 24(25.00)* |  | 85(13.26) | 116(10.61) | 26(12.21) |
| Sometimes | 163(48.22) | 312(51.66) | 49(51.04) |  | 317(49.45) | 608(55.63) | 111(52.11) |
| None | 46(13.61) | 97(16.06) | 23(23.96) |  | 239(37.29) | 369(33.76) | 76(35.68) |
| **Frequency of fried foods intake** |  |  |  |  |  |  |  |
| Often | 21(6.25)** | 48(7.95) | 6(6.25)* |  | 45(7.02) | 70(6.40) | 17(7.94) |
| Sometimes | 269(80.06) | 453(75.00) | 62(64.58) |  | 451(70.36) | 820(75.02) | 156(72.90) |
| None | 46(13.69) | 103(17.05) | 28(29.17) |  | 145(22.62) | 203(18.50) | 41(19.16) |
| **Smoking** ^b^ |  |  |  |  |  |  |  |
| Never smoker | 101(29.97)* | 129(21.39) | 28(29.17)* |  |  |  |  |
| Ex-smoker | 37(10.98) | 71(11.77) | 11(11.46) |  |  |  |  |
| Current smoker | 199(59.05) | 403(66.83) | 57(59.38) |  |  |  |  |
| **Alcohol consumption** ^b^ |  |  |  |  |  |  |  |
| No | 108(32.14) | 194(32.23) | 43(45.26)* |  |  |  |  |
| Yes | 228(67.86) | 408(67.77) | 52(54.74) |  |  |  |  |

^a^ Data was expressed as numbers or percentages and presented as mean with standard deviation of the mean.

^b^ The proportion of smoking and alcohol consumption was too low among women and not given in the table.

**P*<0.05, ** *P*<0.01 denoted significant difference in sociodemographic characteristics and lifestyles between long or short sleep duration and normal sleep duration by gender using *χ*^2^ , Kruskal-Wallis and ANOVA.

Supplementary Table 3 The association between sleep duration and the percentiles of BMI among male participants excluding underweight men ^a b^

| Covariates | OLS | Normal | | | | Overweight | | Obesity | |
| --- | --- | --- | --- | --- | --- | --- | --- | --- | --- |
|  |  | (18.5≤BMI<24.0) | | | | (24.0≤BMI<28.0) | | (BMI≥28.0) | |
|  |  | (q<63.9) | | | | (63.9≤q<93.7) | | (q≥93.7) | |
|  |  | q=2 | q=5 | q=25 | q=50 | q=71 | q=85 | q=95 | q=97 |
| **Sleep duration(hours)** | 0.34(-0.66,1.35) | 0.03(-0.15,0.20) | 0.15(-0.15,0.45) | -0.17(-0.64,0.31) | 0.48(-0.18,1.14) | **0.79(0.23,1.34)** | -0.03(-0.74,0.68) | 0.50(-0.27,1.27) | **1.23(0.81,1.65)** |
| **Squared sleep duration** | -0.03(-0.10,0.04) | -0.01(-0.01,0.01) | -0.01(-0.03,0.01) | 0.01(-0.02,0.04) | -0.04(-0.08,0.01) | **-0.06(-0.10,-0.02)** | -0.01(-0.05,0.05) | -0.03(-0.08,0.03) | **-0.08(-0.11,-0.05)** |
| **Age(years)** | 0.01(-0.03,0.04) | 0.01(-0.01,0.01) | -0.01(-0.01, 0.01) | 0.01(-0.01,0.02) | **0.02(0.01,0.05)** | **0.02(0.01,0.04)** | 0.02(-0.01,0.05) | **0.03(0.01,0.06)** | 0.01(-0.01,0.02) |
| **Education level(years)** | -0.02(-0.16,0.11) | **0.05(0.03,0.08)** | **0.07(0.03,0.11)** | 0.05(-0.01,0.12) | 0.04(-0.05,0.13) | 0.03(-0.05,0.10) | -0.01(-0.11,0.08) | -0.01(-0.11,0.10) | **-0.15(-0.20,-0.09)** |
| **Hours of TV viewing** | -0.02(-0.12,0.08) | -0.01(-0.02,0.02) | -0.01(-0.04,0.02) | -0.02(-0.07,0.03) | -0.04(-0.11,0.02) | **0.07(0.01,0.12)** | **0.17(0.10,0.24)** | **0.20(0.12,0.28)** | **0.13(0.09,0.17)** |
| **Marital status** |  |  |  |  |  |  |  |  |  |
| Unmarried, Divorced, Widowed | 1.00 | 1.00 | 1.00 | 1.00 | 1.00 | 1.00 | 1.00 | 1.00 | 1.00 |
| Married | **1.00(-0.37,2.37)** | **0.24(0.01,0.48)** | 0.32(-0.09,0.73) | **1.12(0.47,1.77)** | **1.23(0.33,2.13)** | 0.60(-0.15,1.36) | 0.77(-0.20,1.74) | **1.76(0.71,2.81)** | **0.71(0.14,1.29)** |
| **Wealth index** |  |  |  |  |  |  |  |  |  |
| Wealthier | **1.41(0.30,2.53)** | -0.10(-0.29,0.10) | -0.14(-0.47,0.19) | **0.60(0.08,1.13)** | **0.89(0.16,1.61)** | **1.17(0.56,1.78)** | **1.09(0.30,1.88)** | 0.29(-0.56,1.14) | **1.49(1.02,1.96)** |
| Medium | 0.76(-0.25,1.77) | -0.10(-0.28,0.07) | -0.06(-0.37,0.24) | 0.28(-0.20,0.76) | **0.85(0.18,1.51)** | **0.89(0.33,1.45)** | **0.99(0.27,1.71)** | **0.95(0.17,1.72)** | **0.50(0.07,0.93)** |
| Poor | 1.00 | 1.00 | 1.00 | 1.00 | 1.00 | 1.00 | 1.00 | 1.00 | 1.00 |
| **Farming frequency** |  |  |  |  |  |  |  |  |  |
| Often | -0.60(-1.56,0.36) | -0.01(-0.18,0.16) | -0.16(-0.45,0.13) | -0.41(-0.86,0.05) | **-1.10(-1.73,-0.47)** | **-1.15(-1.68,-0.62)** | **-1.11(-1.79,-0.42)** | **-1.52(-2.26,-0.79)** | **-1.38(-1.78,-0.97)** |
| Sometimes | -0.52(-1.65,0.62) | 0.04(-0.16,0.24) | 0.03(-0.31,0.37) | 0.02(-0.52,0.56) | -0.53(-1.27,0.22) | -0.37(-0.99,0.26) | -0.65(-1.46,0.15) | **-1.25(-2.13,-0.38)** | **-0.69(-1.17,0.21)** |
| None | 1.00 | 1.00 | 1.00 | 1.00 | 1.00 | 1.00 | 1.00 | 1.00 | 1.00 |
| **Physical activity** |  |  |  |  |  |  |  |  |  |
| Often | -0.49(-1.45,0.46) | **-0.20(-0.37,-0.04)** | -0.15(-0.44,0.13) | -0.34(-0.79,0.11) | -0.27(-0.89,0.36) | -0.36(-0.89,0.16) | -0.55(-1.23,0.13) | **-0.87(-1.60,-0.14)** | **-1.06(-1.46,-0.66)** |
| Sometimes | 0.33(-1.08,1.74) | -0.22(-0.46,0.03) | -0.68(-0.68,0.16) | 0.21(-0.46,0.88) | 1.16(-0.23,2.08) | 0.57(-0.20,1.35) | 0.24(-0.76,1.25) | 0.78(-0.30,1.86) | 0.47(-0.12,1.06) |
| None | 1.00 | 1.00 | 1.00 | 1.00 | 1.00 | 1.00 | 1.00 | 1.00 | 1.00 |
| **Frequency of sweets intake** |  |  |  |  |  |  |  |  |  |
| Often | **-1.16(-2.29,-0.04)** | -0.10(-0.31,0.08) | **-0.51(-0.85,-0.18)** | **-0.69(-1.23,-0.16)** | **-0.99(-1.73,-0.26)** | **-1.33(-1.95,-0.71)** | **-1.65(-2.45,-0.85)** | **-2.23(-3.09,-1.37)** | **-1.78(-2.25,-1.30)** |
| Sometimes | -0.06(-1.07,1.18) | 0.06(-0.14,0.26) | -0.11(-0.44,0.23) | -0.10(-0.63,0.44) | -0.16(-0.89,0.58) | -0.18(-0.79,0.44) | -0.39(-1.19,0.41) | **-1.01(-1.88,-0.15)** | **-0.37(-0.84,0.11)** |
| None | 1.00 | 1.00 | 1.00 | 1.00 | 1.00 | 1.00 | 1.00 | 1.00 | 1.00 |
| **Frequency of fat intake** |  |  |  |  |  |  |  |  |  |
| Often | -0.68(-1.84,0.48) | 0.12(-0.09,0.32) | 0.25(-0.10,0.60) | -0.39(-0.94,0.16) | -0.38(-1.14,0.38) | **-0.71(-1.35,-0.07)** | **-1.06(-1.88,-0.24)** | **-0.99(-1.88,-0.11)** | 0.04(-0.45,0.53) |
| Sometimes | -0.01(-1.07,1.05) | 0.14(-0.05,0.33) | 0.23(-0.08,0.55) | -0.04(-0.55,0.46) | 0.09(-0.61,0.78) | -0.34(-0.93,0.24) | **-0.77(-1.52,-0.01)** | **-1.09(-1.90,-0.27)** | -0.43(-0.87,0.02) |
| None | 1.00 | 1.00 | 1.00 | 1.00 | 1.00 | 1.00 | 1.00 | 1.00 | 1.00 |
| **Frequency of fried foods intake** |  |  |  |  |  |  |  |  |  |
| Often | 0.66(-0.97,2.30) | 0.13(-0.16,0.41) | 0.19(-0.30,0.68) | 0.90(-0.12,1.67) | 0.62(-0.45,1.69) | 0.74(-0.16,1.64) | 0.51(-0.65,1.67) | 0.47(-0.78,1.72) | **0.82(0.13,1.50)** |
| Sometimes | 0.40(-0.62,1.41) | 0.10(-0.08,0.28) | -0.20(-0.50,0.10) | -0.20(-0.68,0.28) | 0.28(-0.38,0.95) | 0.33(-0.23,0.89) | 0.26(-0.46,0.98) | 1.59(0.82,2.37) | **1.61(1.18,2.04)** |
| None | 1.00 | 1.00 | 1.00 | 1.00 | 1.00 | 1.00 | 1.00 | 1.00 | 1.00 |
| **Smoking** |  |  |  |  |  |  |  |  |  |
| Never smoker | 0.18(-0.69,1.04) | **0.33(0.18,0.48)** | **0.34(0.08,0.60)** | 0.24(-0.17,0.65) | 0.26(-0.31,0.83) | 0.48(-0.01,0.96) | 0.22(-0.40,0.83) | **1.01(0.34,1.67)** | **0.89(0.53,1.25)** |
| Ex-smoker | 0.40(-0.78,1.58) | **0.39(0.19,0.60)** | 0.10(-0.25,0.45) | **0.78(0.22,1.33)** | **1.03(0.26,1.80)** | **0.66(0.01,1.31)** | 0.54(-0.30,1.37) | -0.26(-1.16,0.65) | -0.03(-0.52,0.47) |
| Current smoker | 1.00 | 1.00 | 1.00 | 1.00 | 1.00 | 1.00 | 1.00 | 1.00 | 1.00 |
| **Alcohol consumption** |  |  |  |  |  |  |  |  |  |
| No | 1.00 | 1.00 | 1.00 | 1.00 | 1.00 | 1.00 | 1.00 | 1.00 | 1.00 |
| Yes | 0.18(-0.61,0.96) | **0.21(0.07,0.34)** | 0.09(-0.15,0.32) | 0.12(-0.25,0.49) | 0.13(-0.38,0.65) | 0.07(-0.36,0.50) | 0.06(-0.50,0.62) | -0.40(-0.99,0.20) | **-0.49(-0.82,-0.16)** |

^a^ Values were β-estimates (95%CI) of covariates on the percentiles of BMI in the table; Coefficients significant at the 5% level were bold. q denoted the percentiles of BMI.

^b^ The association between continuous sleep time and the percentiles of BMI was assessed using OLS regression and quantile regression model.

Supplementary Table 4 The association between sleep duration and the percentiles of BMI among female participants excluding underweight women ^a b^

| Covariates | OLS | Normal | | | | Overweight | | Obesity | |
| --- | --- | --- | --- | --- | --- | --- | --- | --- | --- |
|  |  | (18.5≤BMI<24.0) | | | | (24.0≤BMI<28.0) | | (BMI≥28.0) | |
|  |  | (q<64.9) | | | | (64.9≤q<94.1) | | (q≥94.1) | |
|  |  | q=2 | q=5 | q=15 | q=50 | q=68 | q=80 | q=97 | q=99 |
| **Sleep duration(hours)** | -0.15(-0.51,0.21) | -0.18(-0.29,-0.06) | -0.22(-0.48,0.03) | -0.03(-0.38,0.32) | 0.04(-0.36,0.44) | -0.18(-0.60,0.24) | 0.12(-0.35,0.59) | -0.18(-0.96,0.59) | **-0.14(-0.24,-0.05)** |
| **Squared sleep duration** | 0.01(-0.03,0.02) | **0.01(0.001,0.018)** | 0.01(-0.01,0.03) | 0.01(-0.02,0.03) | -0.01(-0.03,0.03) | 0.01(-0.02,0.04) | -0.01(-0.04,0.03) | 0.01(-0.05,0.06) | 0.01(-0.01,0.02) |
| **Age(years)** | **0.03(0.02,0.05)** | 0.01(-0.01,0.01) | **0.01(0.001,0.024)** | **0.02(0.01,0.03)** | **0.03(0.01,0.05)** | **0.04(0.02,0.05)** | **0.05(0.02,0.07)** | 0.03(-0.01,0.07) | **0.04(0.03,0.05)** |
| **Education level(years)** | -0.04(-0.09,0.01) | 0.01(-0.01,0.03) | 0.03(-0.01,0.07) | 0.03(-0.02,0.08) | -0.01(-0.06,0.05) | **-0.07(-0.13,-0.01)** | **-0.11(-0.18,-0.04)** | -0.07(-0.18,0.04) | **-0.27(-0.29-0.26)** |
| **Hours of TV viewing** | **0.05(0.01,0.09)** | **0.04(0.03,0.05)** | **0.04(0.01,0.07)** | 0.03(-0.01,0.07) | **0.06(0.01,0.10)** | **0.09(0.05,0.14)** | **0.15(0.09,0.20)** | 0.08(-0.01,0.17) | **0.33(0.32,0.34)** |
| **Marital status** |  |  |  |  |  |  |  |  |  |
| Unmarried, Divorced, Widowed | 1.00 | 1.00 | 1.00 | 1.00 | 1.00 | 1.00 | 1.00 | 1.00 | 1.00 |
| Married | 0.41(-0.15,0.98) | 0.06(-0.12,0.24) | 0.02(-0.40,0.43) | 0.46(-0.11,1.03) | **0.67(0.03,1.31)** | 0.08(-0.60,0.75) | -0.19(-0.94,0.57) | -0.48(-1.73,0.78) | **0.55(0.40,0.70)** |
| **Wealth index** |  |  |  |  |  |  |  |  |  |
| Wealthier | **0.52(0.06,0.97)** | **-0.23(-0.38,-0.09)** | 0.08(-0.25,0.42) | 0.16(-0.29,0.62) | 0.45(-0.06,0.96) | **0.60(0.06,1.14)** | **0.68(0.08,1.28)** | 0.22(-0.78,1.22) | **0.91(0.79,1.03)** |
| Medium | **0.41(0.04,0.78)** | **-0.20(-0.32,-0.09)** | -0.17(-0.45,0.10) | -0.10(-0.47,0.27) | 0.20(-0.21,0.62) | **0.45(0.01,0.89)** | **0.50(0.01,0.99)** | 0.79(-0.03,1.61) | **2.49(2.39,2.60)** |
| Poor | 1.00 | 1.00 | 1.00 | 1.00 | 1.00 | 1.00 | 1.00 | 1.00 | 1.00 |
| **Farming frequency** |  |  |  |  |  |  |  |  |  |
| Often | -0.19(-0.60,0.21) | **0.21(0.08,0.34)** | **0.39(0.09,0.69)** | 0.37(-0.04,0.78) | -0.25(-0.71,0.21) | -0.17(-0.66,0.2) | -0.32(-0.86,0.22) | -0.27(-1.17,0.63) | **-1.06(-1.17,-0.95)** |
| Sometimes | 0.02(-0.47,0.50) | **0.35(0.19,0.50)** | **0.56(0.20,0.92)** | **0.63(0.15,1.12)** | 0.03(-0.52,0.58) | -0.12(-0.70,0.46) | -0.30(-0.95,0.34) | -0.20(-1.27,0.87) | **-1.92(-2.05,-1.79)** |
| None | 1.00 | 1.00 | 1.00 | 1.00 | 1.00 | 1.00 | 1.00 | 1.00 | 1.00 |
| **Physical activity** |  |  |  |  |  |  |  |  |  |
| Often | 0.22(-0.19,0.64) | -0.02(-0.15,0.11) | -0.17(-0.48,0.13) | 0.09(-0.32,0.51) | 0.35(-0.12,0.82) | **0.52(0.02,1.02)** | 0.14(-0.42,0.69) | 0.59(-0.34,1.51) | 1.36(1.24,1.79) |
| Sometimes | 0.13(-0.54,0.80) | 0.02(-0.19,0.23) | 0.01(-0.48,0.51) | 0.04(-0.63,0.72) | -0.02(-0.79,0.73) | 0.40(-0.41,1.20) | 0.99(-0.10,1.89) | 0.06(-1.43,1.55) | -1.28(-1.47,-1.10) |
| None | 1.00 | 1.00 | 1.00 | 1.00 | 1.00 | 1.00 | 1.00 | 1.00 | 1.00 |
| **Frequency of sweets intake** |  |  |  |  |  |  |  |  |  |
| Often | **-0.91(-1.40,-0.41)** | -0.04(-0.19,0.12) | 0.02(-0.34,0.39) | -0.18(-0.68,0.32) | **-0.95(-1.51,-0.38)** | **-1.14(-1.73,-0.54)** | **-1.96(-2.62,-1.29)** | -0.96(-2.07,0.14) | **-0.30(-0.43,-0.16)** |
| Sometimes | -0.43(-0.92,0.06) | **0.33(0.17,0.48)** | 0.29(-0.07,0.65) | 0.14(-0.35,0.63) | -0.53(-1.08,0.02) | **-0.72(-1.31,-0.14)** | **-1.49(-2.14,-0.83)** | 0.09(-0.99,1.18) | -0.04(-0.17,0.09) |
| None | 1.00 | 1.00 | 1.00 | 1.00 | 1.00 | 1.00 | 1.00 | 1.00 | 1.00 |
| **Frequency of fat intake** |  |  |  |  |  |  |  |  |  |
| Often | -0.10(-0.59,0.40) | **0.22(0.06,0.37)** | 0.19(-0.17,0.56) | 0.13(-0.37,0.63) | 0.04(-0.52,0.60) | 0.13(-0.47,0.72) | -0.42(-1.08,0.24) | -0.39(-1.49,0.71) | **1.79(1.66,1.93)** |
| Sometimes | -0.14(-0.45,0.18) | -0.02(-0.12,0.08) | -0.12(-0.36,0.11) | -0.13(-0.45,0.18) | -0.08(-0.44,0.27) | 0.10(-0.28,0.48) | -0.17(-0.59,0.25) | -0.23(-0.93,0.46) | **-0.31(-0.39,-0.22)** |
| None | 1.00 | 1.00 | 1.00 | 1.00 | 1.00 | 1.00 | 1.00 | 1.00 | 1.00 |
| **Frequency of fried foods intake** |  |  |  |  |  |  |  |  |  |
| Often | 0.22(-0.44,0.89) | **0.25(0.04,0.46)** | 0.19(-0.30,0.69) | 0.08(-0.59,0.74) | 0.28(-0.47,1.03) | 0.14(-0.66,0.94) | 0.45(-0.44,1.33) | -0.10(-1.58,1.37) | **0.48(0.29,0.66)** |
| Sometimes | 0.18(-0.20,0.56) | 0.09(-0.03,0.21) | 0.24(-0.04,0.52) | 0.07(-0.31,0.45) | 0.02(-0.40,0.45) | 0.22(-0.24,0.67) | 0.31(-0.20,0.81) | 0.70(-0.14,1.54) | **2.19(2.08,2.29)** |
| None | 1.00 | 1.00 | 1.00 | 1.00 | 1.00 | 1.00 | 1.00 | 1.00 | 1.00 |

^a^ Values were β-estimates (95%CI) of covariates on the percentiles of BMI in the table; Coefficients significant at the 5% level were bold. q denoted the percentiles of BMI.

^b^ The association between continuous sleep time and the percentiles of BMI was assessed using OLS regression and quantile regression model.
